# Neuromotor Noise, Error Tolerance and Velocity-Dependent Costs in Skilled Performance

**DOI:** 10.1371/journal.pcbi.1002159

**Published:** 2011-09-22

**Authors:** Dagmar Sternad, Masaki O. Abe, Xiaogang Hu, Hermann Müller

**Affiliations:** 1 Departments of Biology, Electrical and Computer Engineering, and Physics, Northeastern University, Boston, Massachusetts, United States of America; 2 Research Center for Advanced Science and Technology, The University of Tokyo, Tokyo, Japan; 3 Department of Kinesiology, The Pennsylvania State University, Univeristy Park, Pennsylvania, United States of America; 4 Department of Movement Science, Justus-Liebig University of Giessen, Giessen, Germany; University College London, United Kingdom

## Abstract

In motor tasks with redundancy neuromotor noise can lead to variations in execution while achieving relative invariance in the result. The present study examined whether humans find solutions that are tolerant to intrinsic noise. Using a throwing task in a virtual set-up where an infinite set of angle and velocity combinations at ball release yield throwing accuracy, our computational approach permitted quantitative predictions about solution strategies that are tolerant to noise. Based on a mathematical model of the task expected results were computed and provided predictions about error-tolerant strategies (Hypothesis 1). As strategies can take on a large range of velocities, a second hypothesis was that subjects select strategies that minimize velocity at release to avoid costs associated with signal- or velocity-dependent noise or higher energy demands (Hypothesis 2). Two experiments with different target constellations tested these two hypotheses. Results of Experiment 1 showed that subjects chose solutions with high error-tolerance, although these solutions also had relatively low velocity. These two benefits seemed to outweigh that for many subjects these solutions were close to a high-penalty area, i.e. they were risky. Experiment 2 dissociated the two hypotheses. Results showed that individuals were consistent with Hypothesis 1 although their solutions were distributed over a range of velocities. Additional analyses revealed that a velocity-dependent increase in variability was absent, probably due to the presence of a solution manifold that channeled variability in a task-specific manner. Hence, the general acceptance of signal-dependent noise may need some qualification. These findings have significance for the fundamental understanding of how the central nervous system deals with its inherent neuromotor noise.

## Author Summary

It is widely recognized that variability or noise is present at all levels of the sensorimotor system. How the central nervous system generates functional behavior with a sufficient degree of accuracy in the face of this noise remains an open question. This is specifically relevant when the motor task is redundant, i.e., where many *different* executions can achieve the *same* task goal. Using an experimentally controlled throwing movement as model task we examined how humans acquire movement strategies that are tolerant to intrinsic noise. Based on a new computational approach that parses variability based on an analysis of task redundancy, we tested two hypotheses: 1) Subjects are sensitive to noise and seek solutions that are tolerant to this noise. 2) Subjects avoid solutions with high velocities and the costs associated with high velocities. Analysis of the distributional properties of variability in two experiments revealed that humans select those strategies that maximize error-tolerance. These findings have significance for fundamental understanding of the central nervous system and for learning in the context of rehabilitation.

## Introduction

Decrease of error and its variability as a consequence of practice is a widely recognized indicator of skilled performance and improvement. More recent studies have tried to look beyond pure outcome measures and examined the variability at different stages in movement generation, for example during the planning stage [1], during the execution of movements [2], [3], and in the processing of sensory estimates [4]. Such variability or noise is the consequence of many processes at all spatiotemporal levels of the sensorimotor system arising, for example, in signal propagation due to synaptic fluctuations that affect the regularity of spike trains, or in the transduction of a continuous signal into discrete spike sequences [5]. This variability has been shown to depend on the signal amplitude, for example the magnitude of contractile force or velocity. It has become widely accepted that subjects aim to minimize signal-dependent noise [6], [7].

Over recent years sensorimotor noise and its role in motor control has received increasing attention from several lines of study. For example Trommershäuser, Maloney and colleagues have focused on rapid pointing tasks where variability in pointing accuracy was analyzed with respect to different penalties and rewards [8], [9]. Several studies have shown that human performers take their variability and the risk induced by their own uncontrolled variability into account. Their research has been guided by the framework of decision theory and emphasized the cognitive decision making and planning when performing a motor task. Van Beers and colleagues have looked at variability of reaching tasks as an entry to understand visual and proprioceptive information contributing to motor solutions [3], [10]. Variability and noise is also central in the work on stochastic optimal feedback control by Todorov and colleagues and this computational approach has been applied to increasingly more diverse tasks [11], [12], [13], [14]. A recent study by Nagengast, Braun, and Wolpert highlighted that this optimal control framework may need to be differentiated to address inter-individual differences in risk attitudes, i.e., individuals' preferences to deal with risk and penalties [15].

Our research on variability and noise complements and extends these lines of research in several aspects. The present study examines performance of a motor skill where redundancy in the task presents different opportunities for dexterous performance. To be explicit, redundancy in the task permits that an infinite set of executions leads to the same result, both for zero-error solutions but also all other non-zero task solutions. This redundancy has been frequently illustrated in a multi-joint pointing movement where an infinite number of joint-angle combinations lead to a given accuracy in the endpoint position. In our single-joint throwing task an infinite set of states at the moment of ball release, position and velocity of the arm movement, leads to zero-error performance. However, not all solutions are the same with respect to risk and sensitivity to error. Mathematical analysis of the task's redundancy presents the platform for an analysis of subjects' variability over repeated executions.

Repetitions of the “same” movement will lead to variations not only as a consequence of the ever-present noise in the sensorimotor system but also due to the geometry of the null space of the task that endows different solutions with different degrees of tolerance or sensitivity to errors. Hence, the observed variability is not necessarily random, but rather its distribution may express strategies of the central nervous system. Our analysis will focus on distributional aspects of execution with respect to the geometry of the null space or solution manifold determined by the task. Related approaches such as decision-theoretic, optimal control models, or the *U*n*C*ontrolled *M*anifold (UCM) method have provided support that the variability over multiple repetitions is structured. For example the *U*n*C*ontrolled *M*anifold (UCM-) approach [16], frequently applied to variability in joint space with respect to its mean endpoint position or force contributions of fingers with respect to summed force output has provided support that variability in directions parallel to the null space is larger than variability orthogonal to it [17]. Interestingly, this structured variability is also the consequence of the optimization of control cost in the optimal feedback control models [13]. While the goal of the UCM-analysis resembles our approach, some critical differences exist in how the problem is posed, how variability is analyzed and, consequently, the obtained result [18], [19]. The present study illustrates our approach and how it permits specific predictions about strategies with a view to a desired task result.

One critical difference between our approach and the UCM-method and optimal feedback control is that they have only focused on the covariance structure of the distribution with respect to a solution manifold. In contrast, our work developed an analysis of variability that differentiates between three different contributions to optimal task performance. This TNC-method allows the quantitative analysis of *Tolerance*, *Noise* and *Covariation* [19], [20], [21], [22]. The component *Noise* is straightforward and refers to the amplitude of the random distribution. *Covariation* is indicated when the data are aligned with the solution manifold, conceptually identical to what the UCM method and also optimal feedback control focused on. Our quantification, however, does not rely on the analysis of the covariance structure which is stricken with sensitivity to coordinates [18].

Unique to our analysis is the concept of *Tolerance* that evaluates movement strategies with respect to the error that deviations from the ideal solution incur, i.e., tolerant solutions are least sensitive to error and perturbations. It should, however, be pointed out that this concept is not equivalent to local sensitivity as *Tolerance* is defined over the neighborhood defined by the subject's variability. Note also that maximizing *Tolerance* is different from the goal of “maximizing hit rate” in a single trial by processing feedback to decrease error. Rather, it is defined over a set of performances and quantifies to what degree subjects are sensitive to their own errors and take predicted cost of a set of trials into account. Previous experiments have shown how *Tolerance* is the first component that is reduced with practice [20], [22]. The present study shows how a task analysis can generate predictions that permit direct evaluation of whether subjects seek out error-tolerant strategies, i.e., strategies that allow maximum variability at the execution level but with minimal penalty in the result.

To this end we examine a throwing task called skittles in which a subject throws a ball suspended to a vertical post to hit a target skittle at the other side of the post. The task is redundant such that an infinite set of variations can have the same result. In the experimentally controlled task two *execution variables*, angular position and velocity at release of the ball, fully determine its *result variable*, the ball's trajectory and its error from hitting the skittle. The key characteristic is that the number of execution variables is larger than the number of result variables; hence, an infinite number of angle and velocity combinations can lead to the same distance error. The results with zero error form a set called the *solution manifold*. Hence, this task is representative for any goal-oriented skill where a redundant number of execution variables fully determine the result.

This study examined the hypothesis whether subjects are sensitive to their motor variability and find error-tolerant solutions that minimize the effect of this variability on their performance result (*Hypothesis 1*). Yet, in the present task successful throwing actions can be executed with a large range of different velocities. As it is commonly assumed that higher velocities are associated with higher costs, such as signal-dependent noise or some form of energy or effort, it can also be hypothesized that subjects seek solutions with the lowest possible velocity (*Hypothesis 2*). Two experiments with different task configurations will test these two hypotheses.

## Methods

### Ethics Statement

Prior to data collection, subjects were instructed about the experimental procedure upon which they signed an informed consent form in agreement with the Institutional Review Board of the Pennsylvania State University.

#### Participants

A total of 18 graduate students (11 male, 7 female, 22 to 30 years of age) from the Pennsylvania State University volunteered to participate in the two experiments (9 participants in each). They all reported themselves to be right-handed and had no neurological disorders. They were informed about the purpose of the experiment, but were naive about the nature of the manipulations in the experiment.

### The Model Task

**Figure 1:**
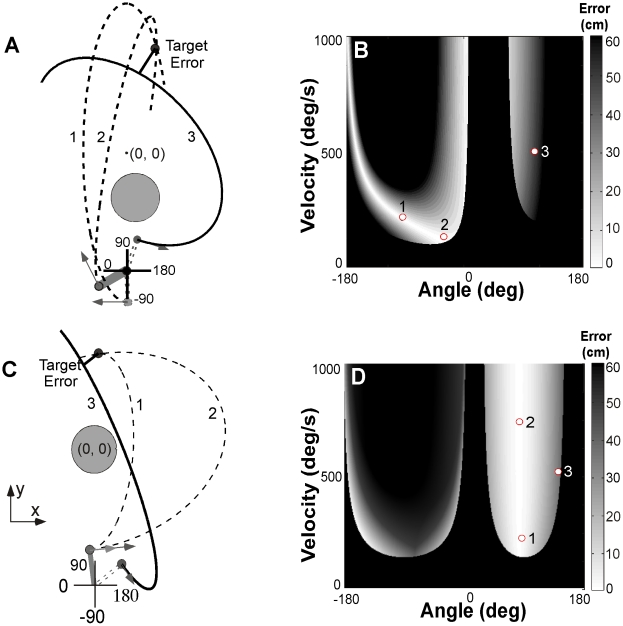
Workspace, execution space and solution manifold. **A:** Workspace with the position of the center post and target skittle in Experiment 1. Two ball trajectories exemplify how different release variables can lead to the same result with zero error (trajectory 1, 2, dashed lines). Trajectory 3 shows a trajectory with non-zero error. **B:** Workspace with center post and target as used in Experiment 2. Three select trajectories exemplify the redundancy of solutions as in panel **A**. **C:** Execution space and solution manifold of target and center post configuration in Experiment 1. White denotes zero-error solutions, increasing error is shown by increasingly darker grey shades, black denotes a post hit. The release variables of trajectory 1 and 2 correspond to points 1 and 2 on the solution manifold, the variables of trajectory 3 correspond to the point 3 in a grey-shaded area (error  = 30cm). **D:** Corresponding execution space and solution manifold. The three points correspond to the three trajectories of panel **B**.

The task for the present study is similar to the game skittles or tetherball where the person throws a ball suspended as a pendulum around a pole to hit a target at the opposite side of the post. The trajectory of the ball is fully determined by the angular position and velocity at release of the ball and the mathematical relationship is modeled using basic mechanics [22]. After release, the ball trajectory describes an elliptic trajectory around a center post from which it is suspended (centripetal force field). Performance results or errors are quantified by the minimal distance between the ball trajectory and the target skittle. Figures 1A and 1B shows a top down view as subjects saw it during the two experiments, respectively. A successful hit with zero error meant that the center of the ball went through the center of the target. In case they did not hit the skittle, the error was calculated as the minimum distance between the trajectory and the center of the target.

In both examples two of the three trajectories illustrate how different combinations of the two execution variables can lead to the same result (error  = 0), i.e., the task is redundant. The redundancy relation between execution and result variables is captured in the execution space (Figure 1C and 1D) where every throw, defined by the variables angle and velocity, corresponds to one point. Different levels of success, quantified in the error, are displayed by different grey shades. Perfect hits with zero error are displayed in white and form the one-dimensional solution manifold; solutions with increasing error are shown by increasingly darker grey shades; black denotes a post hit. As the two constellations exemplify, different positions of the target and the center post create very different execution spaces and solution manifolds.

### Experimental Set-Up

**Figure 2:**
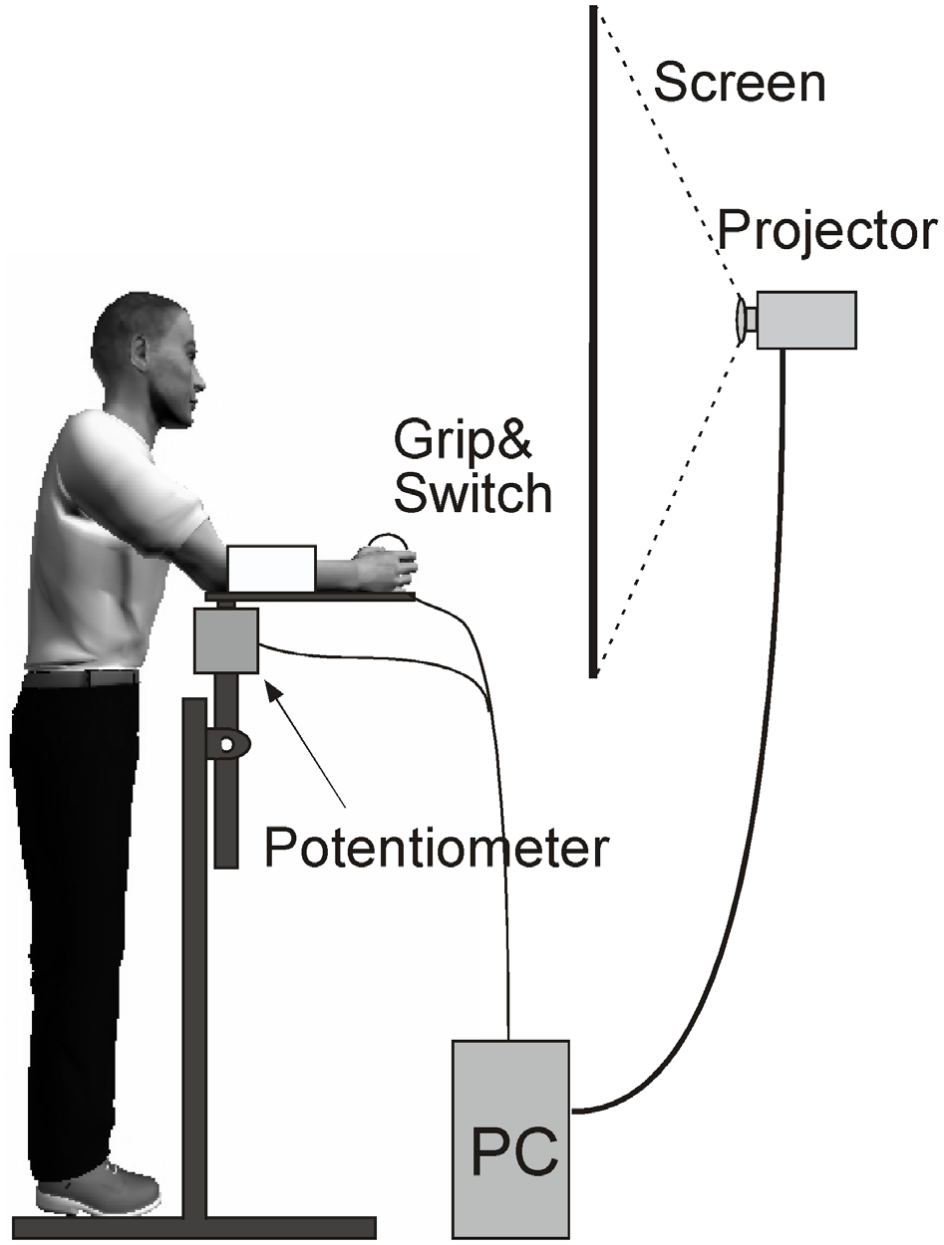
Experimental setup. Participants stand in front of the setup with their forearm resting on the horizontal lever arm. The rotation of the arm is recorded by the potentiometer, when the finger opens the contact switch the ball in the virtual simulation is released. Online recordings of the arm movements are displayed on the projection screen.

Participants stood in front of a back projection screen operating a lever arm that simulated the throw (Figure 2). The height of the lever was adjustable for each person so that his/her forearm was placed horizontally with the elbow joint aligned with the axis of rotation. At the distal end of the manipulandum, the participant grasped a ball and closed a contact switch with his/her index finger. Extending the index finger corresponded to opening the grasp to throw the ball; this opened the switch and triggered the release of the ball on the visual display. The rotation of the manipulandum was measured by a potentiometer (Vishay Spectrol, CA) with a sampling rate of 650 Hz. The participant could stand to the right or left of the vertical fixation, throwing in clockwise or counterclockwise direction, depending on the task.

The visual display (60 Hz update rate) was presented on a back projection screen (1.80 m×1.40 m) positioned 0.60 m in front of the participant. On the screen he/she saw the virtual lever arm moving in real-time that threw a ball to hit a target skittle on the other side of the center post. The ball trajectory was computed from the online measurements of angular position and the numerically differentiated velocity at release according to the model described in [22]. The ball's trajectory was displayed for 1 s after release, which was sufficient to provide visual feedback about the success of the throw.

The data acquisition and the visual display were programmed in Visual C++ (Microsoft, v6.0); the virtual display was implemented by Open GL Graphics (Silicon Graphics, v1.2).

### Experimental Procedure and Design

Participants were instructed to hit the target with the virtual ball as accurately as possible. After a self-timed short break subjects initiated the next trial. Typically, one trial including the break between trials lasted approximately 6 s. The throwing movement itself lasted approximately 350 ms. In Experiment 1 subjects performed three sessions, each consisting of three blocks with 60 trials in one block, yielding a total of 540 trials; in Experiment 2 each subject performed five sessions, giving a total of 900 trials. Between each block, participants rested for a few minutes. The total duration of each session was approximately 15 min. The sessions were collected on three and five consecutive days, respectively. In session 1 participants were instructed to try different release angles and release velocities to find successful strategies that achieved reliable solutions. In the subsequent sessions participants were instructed to no longer explore but to continue with the strategy that had proven most successful. Note, by strategy we do not mean that subjects necessarily have to repeat a single solution, but rather stay in the ballpark of solutions. They were encouraged to fine-tune their performance and avoid hitting the center post as they would receive a large penalty.

A third control experiment was performed to examine whether performance of the throwing action without a target resulted in different levels of variability that depended on the release angle or velocity. In this Experiment 3 six subjects were asked to perform the same throwing movements, only that there was no target skittle. The instruction to the subjects was to perform the throwing movements at their preferred velocity but also at two higher velocities and two lower velocities than preferred. The only constraint was to avoid hitting the center post. Subjects performed five blocks of 25 trials each, each block with one of the five instructed velocities. The sequence of blocks was randomized across subjects.

### Target Configurations with Execution Space and Solution Manifold

In Experiment 1 subjects saw the workspace as shown in Figure 1A with the target located at coordinates (35, 125 cm) and the center post with a radius of 33 cm located off center at (10.5, −60 cm). The target skittle (radius 1.50 cm) was located at (35, 125 cm). The ball radius was 2.50 cm. Figure 1C represents the associated execution space with the nonlinear solution manifold (error  = 0 cm), shown in white. Although each solution on the manifold is equivalent, different locations on the solution manifold have very different sensitivity or tolerance to errors, as illustrated by the changing curvature of the result function adjacent to the solution manifold. For reference, if the trajectory only touched the target, the error was 4 cm. If the ball hit the center post, the trial was penalized with the relatively large error of 60 cm, shown in black.

The execution space for Experiment 1 was so designed that successful solutions could take on a relatively large range of release angles and the curvature at the solution manifold showed a pronounced change: smaller release angles showed higher tolerance to error – the curvature of the result function was shallow. Additionally, the most tolerant region transitioned discontinuously to one associated with large penalty – strategies that resulted in post hits. Solutions that allowed for a relatively large dispersion were adjacent to solutions that are penalized heavily – risky strategies.

In Experiment 2, subjects saw the workspace as shown in Figure 1B. The target was located at the coordinates (5.0, 105.8 cm) and the post was slightly smaller (radius 25 cm) but centered at the origin (0, 0). Figure 1D represents the associated execution space with the solution manifold that was approximately parallel to the velocity dimension, i.e. execution strategies were only little sensitive to velocity. This sensitivity or tolerance to variations in angle increased for higher velocities, although the gradient was relatively small. Importantly also, the solution with the lowest velocity was adjacent to the penalized post hits and therefore posed a risky strategy.

### Hypothesis Testing

To test the two hypotheses we performed simulations to render quantitative predictions for tolerant solutions. For *Hypothesis 1* the error-tolerance *T* of all possible executions, i.e. angle-velocity (α, *v*) pairs, was computed. As *Tolerance T* is defined for a given distribution of data, we used the average standard deviations of all subjects in the present two experiments, determined a posteriori from all participants as a representative distribution. While an estimate of variability based on previous experiments would have served this purpose, a more accurate estimate was obtained from the actual standard deviations. In Experiment 1 these standard deviations were *SDα*  = 11.70 deg and *SDv*  = 40.49 deg/s, as determined from the grand average over sessions 2 and 3. In Experiment 2, these standard deviations were *SDα*  = 9.44 deg and *SDv*  = 70.38 deg/s, calculated over sessions 2 to 5. These dispersions defined the size of the neighborhood for each location in execution space 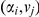
where *i*  = 1, 2, …360 denotes one bin in the angle dimension, and *j*  = 1, 2, … 360 denotes one bin in the velocity dimension (see Text S1 for more detail). For each 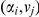 *Tolerance* was calculated as a weighted average error, 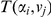. The weights over this matrix neighborhood were taken from a bivariate Gaussian distribution. 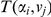 was assigned the weighted average (see Text S1 for details).

To translate these *Tolerance* values into an estimate of probability by which subjects chose this strategy, 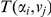 was transformed by an exponential function, the softmax activation function, to obtain the expected results *E(R)* for each result 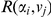 [23]:
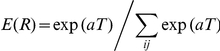



The denominator is a normalization factor that scaled the values of *E(R)* to the range [0,1]. The parameter *a* was fitted based on the pooled data distributions using least square fits. This transformation paid tribute to the fact that the subjects' probability of choosing a given strategy did not scale linearly with the expected *Tolerance*. Rather, solutions with small error were given high preference, while solutions with intermediate and large errors were much less preferred and thereby less probable (see Text S1 for details).

For Hypothesis 2 – predicting preference for the velocity-sensitive strategy – the initial *Tolerance* estimates for each 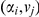 were also transformed by the softmax activation function. However, this transformation included an additive term that evaluated velocity *v*:
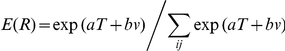



Analogous to Hypothesis 1, the two parameters *a* and *b* were fitted to the pooled data distributions using least square fits (see Text S1 for details).

**Figure 3:**
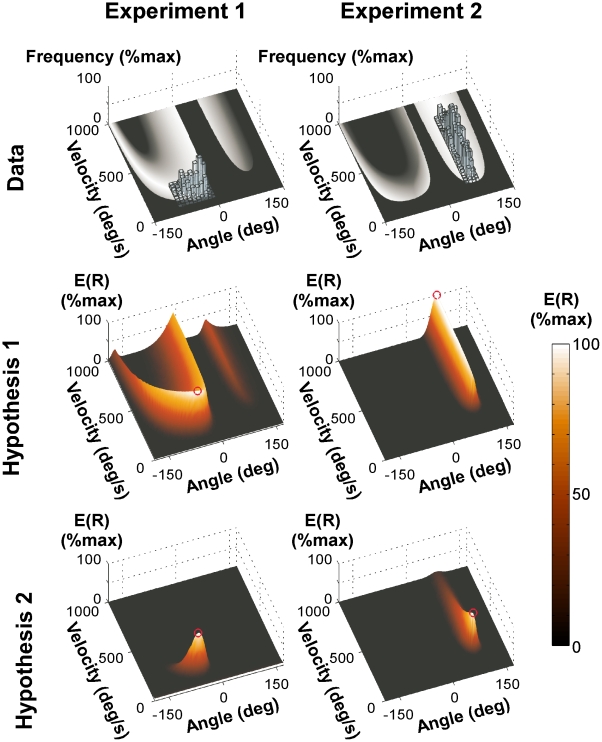
Histograms of all data and predictions for Hypotheses 1 and 2 for both experiments. **A, B**: Histograms of all subjects' trials plotted onto the execution space of Experiment 1 and 2 (see Figure 1B and D). The data are plotted onto a grid of 36x36 bins on the execution space. **C, D**: Simulation of Hypothesis 1: The vertical dimension represents the expected result *E(R)* calculated as the Gaussian weighted averages over a matrix of execution variables transformed by the softmax function. The most error-tolerant solution with maximum *E(R)*, shown by the red circle, is at *α* = −44 deg and *v* = 161 deg/s. In Experiment 2 error-tolerant solutions quantified as expected result *E(R)* are at an angle *α* = −82 deg, the optimal strategy for *E(R)* is at the highest velocity *v* = 1000 deg/s. **E, F**: Simulation of Hypothesis 2: The expected result *E(R)* has its optimal value at the minimum velocity *α* = −29 deg and *v* = 122 deg/s. In Experiment 2 *E(R)* shows its maximum value at *α* = 83 deg and *v* = 142 deg/s.

Figure 3 illustrates the data distributions in both experiments and the two quantitative predictions for both experimental target constellations. The top two panels show the histograms of all subjects' data pooled, plotted on the respective execution space (compare to Figure 1C and D). These histograms provided the reference for parameterizing the softmax function for the quantitative predictions. The two middle panels show the predictions of Hypothesis 1. For Experiment 1 the maximum value of *E(R)* with highest *Tolerance* was at *α* = −44 deg and *v* = 161 deg/s (indicated by the red circle). For Experiment 2 the different target constellation rendered the maximum of *E(R)* and highest *Tolerance* at the highest velocity for the given range: *α* = −82 deg and *v* = 1000 deg/s. It should be pointed out that the slope was very gradual and for higher velocities the change in *E(R)* was very small. Note that the exponential transformation decreased *E(R)* for intermediate or lower result values, thereby enhanced the contrast between good and less good solutions. The two bottom panels of Figure 3 show the simulation results for Hypothesis 2: For Experiment 1 the predicted optimal strategy was at *α* = −29 deg and *v* = 122 deg/s. While this optimum was close to the one of Hypothesis 1, the gradient around it was much steeper. For Experiment 2, the strategy with minimum velocity was at *α* = 83 deg and *v* = 142 deg/s. In this experiment, the two hypothesized solutions were at opposite ends of the manifold.

### Statistical Analyses of Data Distributions

To evaluate the subjects' distributions several analyses were conducted. First, to visualize each individual's distribution in execution space the covariance matrix of the execution variables was calculated and shown by its 95% confidence ellipse. Three parameters described the confidence ellipse: 1) the mean of release angle and velocity determined the center of the ellipse, 2) the eigenvectors were calculated to determine the orientation of the ellipse, and 3) the square roots of the eigenvalues determined the size of the semi-major and semi-minor axes of the ellipse. Given that the confidence ellipse required a large number of samples the data of all sessions, except session 1 were pooled. To test the two hypotheses, a first simple test evaluated how many confidence ellipses, i.e., subjects, overlapped with the predicted optimal value of Hypotheses 1 and 2. This resulted in a simple count that was compared with an expected frequency derived under the assumption that there was no preference for any specific solution.

A second more thorough test examined each individual's distribution and compared it with the hypothesized distribution at the respective location in execution space. To this end, the trial distributions of each subject (360 trials in Experiment 1 and 720 trials in Experiment 2) were presented in execution space in a matrix of 5x5 cells centered on the mean angle and velocity; the matrix size was determined by the individual's standard deviations. The number of cells for the matrix was based on the recommended √*n*, which suggested 18 cells for Experiment 1 and 27 cells for Experiment 2. To facilitate comparison of results for Experiments 1 and 2 we chose 25 cells, or a 5x5 matrix for both. The frequency distributions of the data were compared with the predictions for *E(R)* from Hypotheses 1 and 2 using likelihood estimates. Given that the predictions for Hypothesis 2 contained two fitting parameters, it was evident that Hypothesis 2 had to fare better. Hence, for the comparison of the two nested model fits, we applied the Akaike Information Criterion AIC that evaluated the goodness of fit in the face of different parameters.

## Results

### Experiment 1

#### Performance improvement

**Figure 4:**
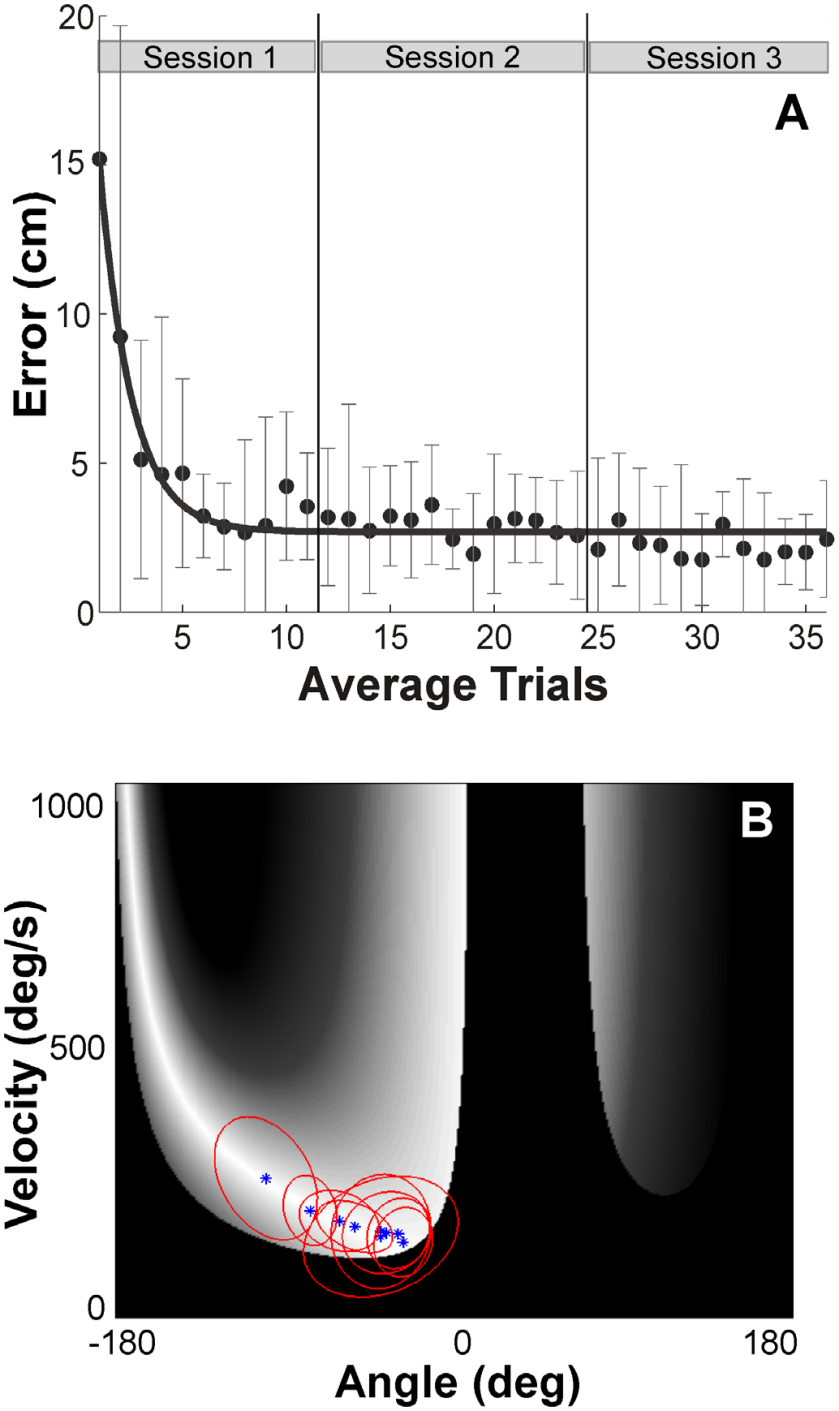
Descriptive results of Experiment 1. **A:** Time series of errors (median and interquartile range) averaged across 9 participants. The trials were also averaged such that for every non-overlapping series of 15 trials the median was plotted with the corresponding interquartile ranges shown by the error bars. The line represents an exponential fit to highlight the time course. **B:** Distribution of trials of individual participants in sessions 2 and 3 plotted in execution space. The 360 trials of each of the 9 participants are represented by the 95% confidence ellipses.

A first evaluation of the data examined how performance errors decreased with practice over all trials across all sessions (Figure 4A). For each estimate the data of 9 participants were pooled over 15 trials and each data point represents the median with its interquartile ranges shown by the error bars. Medians were displayed because the discontinuously high penalties for the post hits would have unduly skewed the means. The line represents an exponential fit to highlight the time course of the change with practice. It can be seen that after large errors in the first half of session 1 participants reached a relatively constant level of performance that they maintained throughout the rest of the experiment. The initially large errors were partly due to the fact that participants were instructed to explore different strategies until they found a strategy that achieved good hitting success. In the subsequent sessions they were instructed to continue and fine-tune their performance. Hence, the average change in error in session 1 was large (12.02 cm) compared to session 2 (0.56 cm) and session 3 (0.34 cm). Given this qualitative difference in the amount of improvement, the data from session 1 were excluded from subsequent analyses.

#### Pooled distributions

The next focus was on the skilled performance that participants had reached in sessions 2 and 3. Returning to Figure 3A the plotted histograms show the trials of sessions 2 and 3 pooled from all 9 participants plotted in execution space; the space was divided into a 36x36 grid (defined over the entire execution space). The distribution was clearly non-uniform and clustered around a mode at an angle of −36 deg and velocity 136 deg/s. None of the zero-error solutions at higher velocities and larger angles were used. Instead, this mode was close to the maximally tolerant *E(R)* as predicted by Hypothesis 1 but also close to Hypothesis 2 (Figure 3C, E). Note that the highest frequency of trials was also close to the locations with the high penalty, i.e., executions that lead to a post hit (shown in black). In fact, a non-negligible number of trials were in the post hit region. Hence, the pooled data seemed to favor maximizing tolerance and minimizing velocity while accepting some risk.

#### Individual distributions

Figure 4B illustrates the distributions of the 360 trials for each of the 9 participants separately by their mean and 95% confidence ellipses. The figure demonstrates that individuals showed overall smaller distributions along the solution manifold with some subjects close to the discontinuity and others well away from the risky strategy. Despite the inevitable disparity across individuals, the maximum *E(R)* predicted by Hypothesis 1 was within the confidence ellipses of 7 of the 9 participants. If there was no preference, all solutions within the angle range of −165 deg to 0 deg should have been chosen with equal probability. Given that the average radius of the 9 confidence ellipses was approximately 25 deg, an ellipse covered approximately 30% of the range of all solutions. The probability that such a confidence interval contains the maximum *E(R)* is 30% under the assumption that the centers of the ellipses are uniformly distributed across all possible solutions. A binominal test revealed that the observed distribution 7/2 is only expected with a probability of *p* = 0.004 under these assumption.

#### Hypothesis testing

**Figure 5:**
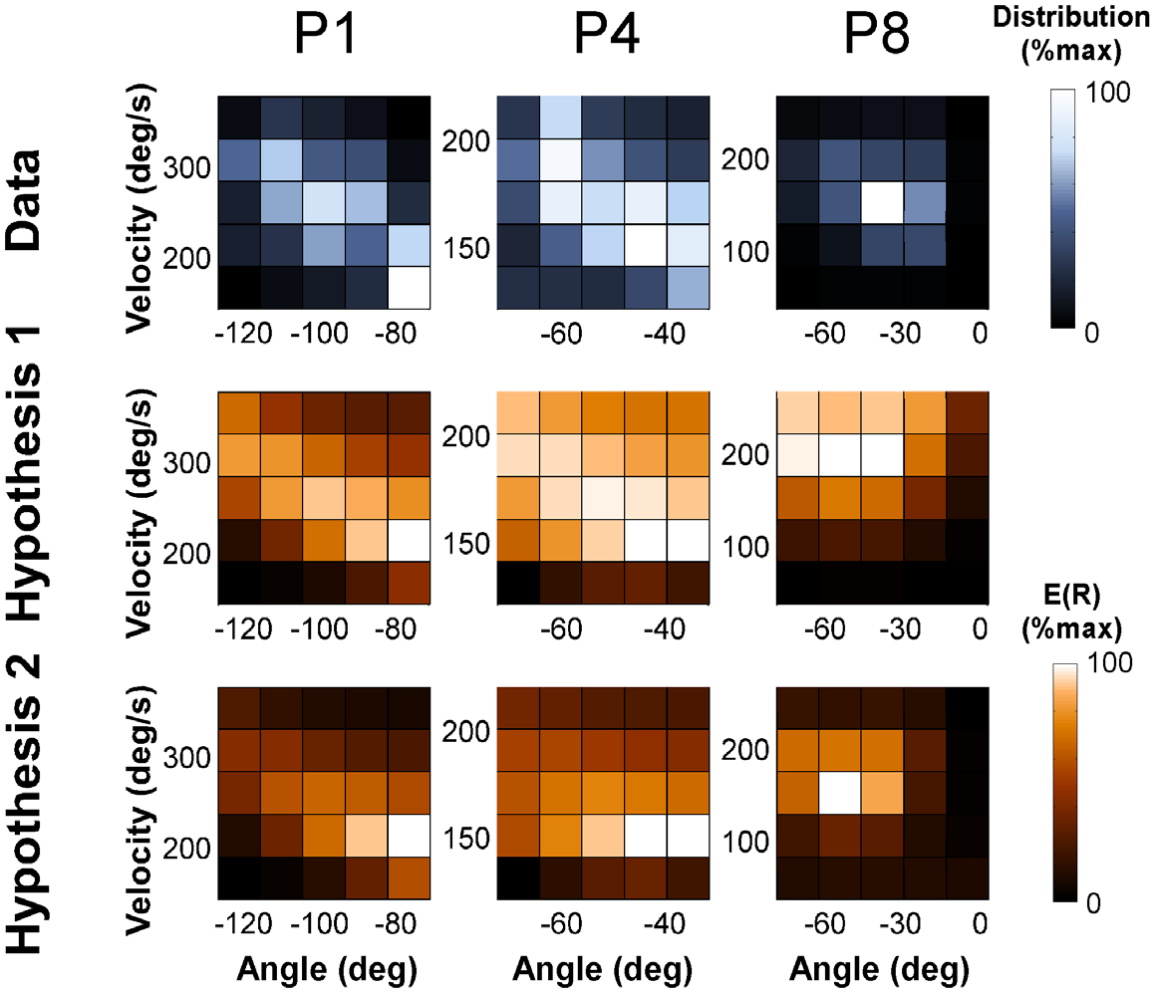
Two-dimensional histograms of two representative individuals' data and the corresponding hypothesized distributions for Experiment 1. The left panel shows the trial frequency, the middle panel shows the expected result *E(R)* of Hypothesis 1, the right panel shows the predicted distribution of Hypothesis 2. As the units of the three distributions are different they were all normalized to the range between 0 and 1. Note that the black color codes the lowest value and should not be mistaken for the high-penalty regions in Figure 3.

In order to test the two hypotheses, the data of each individual were correlated with the predicted values. Figure 5 illustrates the two-dimensional correlation analyses with three representative participants (P1, P4 and P8). The histograms of the data are plotted in blue shades in the top row and the histograms of the expected results *E(R)* for Hypotheses 1 and 2 are displayed in orange shades, consistent with Figure 3, in the middle and second and third row. (Note that the dark shades are not to be confused with the post hits in Figure 3.) Importantly, the expected results *E(R)* were re-calculated on the basis of the individual's standard deviations (the simulations for the hypotheses used the subjects' average standard deviations). Hence, the predictions for the two hypotheses were optimized for each individual and differed accordingly. Each individual's data was tested against *E(R)* at the respective location in execution space. For example, Participant 1′s data distribution shows a diagonal orientation which mirrors the predicted orientations by Hypotheses 1 and 2. (For better comparison the participants were numbered according to their mean release angle to allow visual comparison with the ellipses from left to right in Figure 4A.) Similar tendencies are seen in P4 and P8 whose predicted distributions differ significantly from P1 due to their different location in execution space (compare with Figure 4A). Qualitatively, both hypotheses approximate the data fairly well with a slight advantage for Hypothesis 2.

The statistical results of all participants are summarized in Table 1. The table shows the log likelihood fits LL for all 9 participants together with the fitted parameters *a* and *b* of the softmax function for each participant and the Aikaike Information Criterion AIC. The comparison of the two fits for Hypotheses 1 and 2 shows slightly better values for Hypothesis 2 for all participants. However, this is inevitable given that the model for the two hypotheses were nested such that *E(R)* for Hypotheses 2 extended the model for Hypothesis 1. Hence, the only reliable basis for comparison is AIC. Lower AIC values indicate a better fit, discounting the fact that Hypothesis 2 had one more parameter. Using this criterion, the fits for Hypothesis 1 were better for all 9 participants.

**Table 1:** Results of likelihood analyses testing Hypotheses 1 and 2.

Experiment 1							
	Hypothesis 1			Hypothesis 2			
Participant	a1	LL	AIC	a2	b	LL	AIC
1	106	−3.05	8.11	114	7000	−3	9.99
2	168	−3.12	8.25	200	6400	−3.05	10.1
3	152	−3.13	8.26	188	3600	−3.1	10.2
4	100	−3.18	8.36	140	3000	−3.16	10.31
5	22	−3.14	8.28	72	12000	−2.99	9.98
6	26	−3.14	8.27	66	8800	−3.03	10.06
7	18	−3.18	8.36	60	9400	−3.05	10.11
8	22	−3.08	8.16	66	13800	−2.88	9.76
9	18	−3.13	8.27	44	7200	−3.07	10.14

Experiment 2							
	Hypothesis 1			Hypothesis 2			
Participant	a1	LL	AIC	a2	b	LL	AIC
1	2500	−3.07	8.15	2500	0	−3.07	10.15
2	1200	−3	7.99	1200	22800	−2.86	9.72
3	1400	−3.15	8.29	1450	7800	−3.13	10.25
4	1000	−3.05	8.10	1000	0	−3.05	10.1
5	1000	−2.98	7.96	1100	10800	−2.92	9.84
6	1000	−2.95	7.90	1000	2400	−2.95	9.89
7	1050	−3.05	8.09	1050	0	−3.05	10.09
8	800	−3.05	8.09	800	1500	−3.04	10.09
9	150	−3.12	8.25	150	0	−3.12	10.25

Likelihood analyses testing Hypotheses 1 and 2 for Experiments 1 and 2 (*LL* refers to the log-likelihood estimate). These analyses compared the 2D frequency distribution of each individual on a 5x5 matrix with the expected result *E(R)* from Hypotheses 1 and 2 on the same matrix (see details in the text). The participants are numbered according to their mean release angle in Experiment 1 and mean release velocity in Experiment 2 to facilitate visual comparison with their data shown in Figures 4B and 5B, respectively.

### Experiment 2

#### Performance

**Figure 6:**
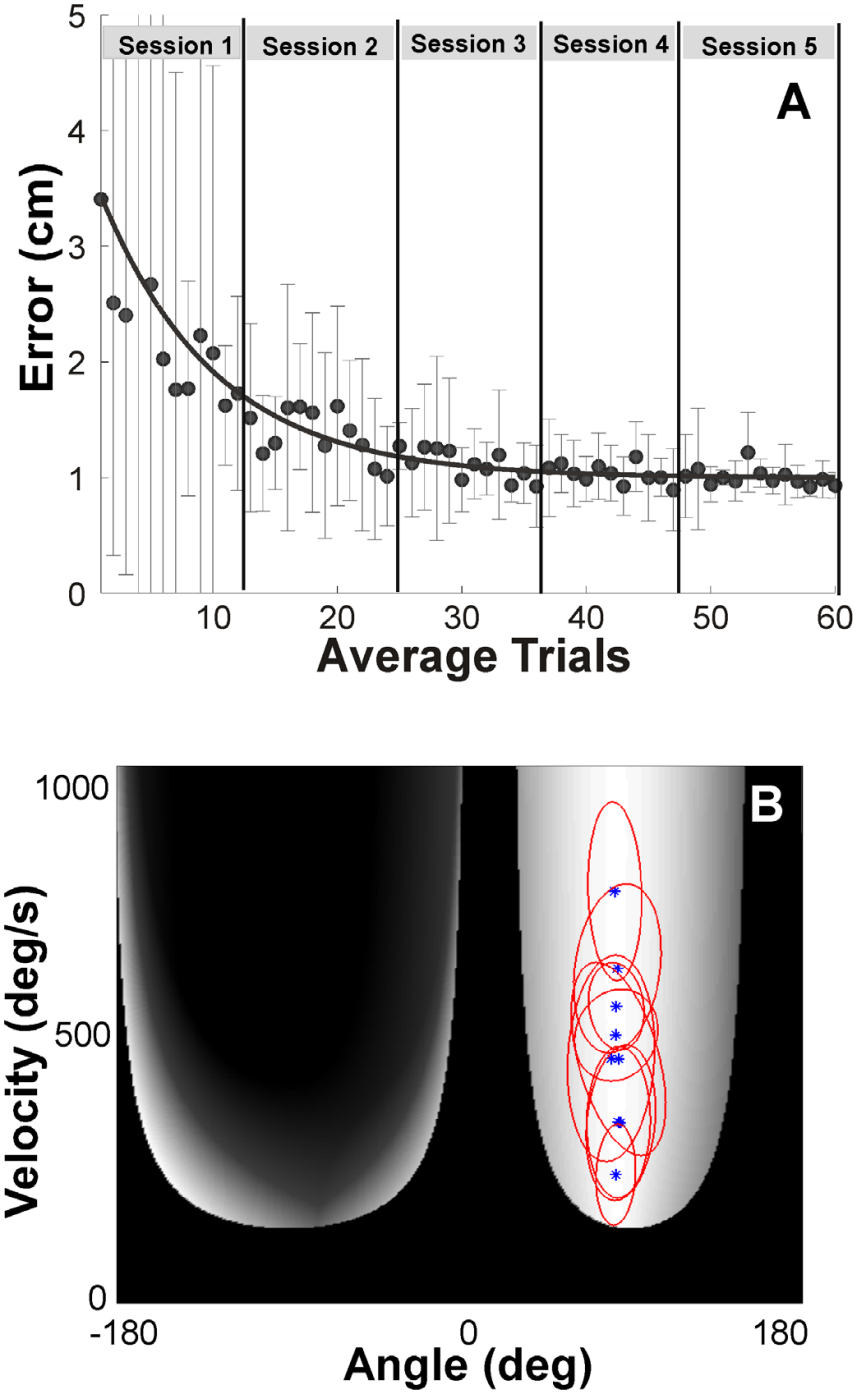
Descriptive results of Experiment 2. **A:** Time series of errors over trials (median and interquartile range). The errors were averaged over 9 participants. The trials were also averaged such that for every non-overlapping series of 15 trials the median was plotted with the corresponding interquartile ranges shown by the error bars. The line represents an exponential fit to highlight the time course. **B:** Distribution of trials of individual participants in sessions 2 to 5 in execution space. The 720 trials of each of the 9 participants are represented by the 95% confidence ellipses.

Figure 6A shows the medians and interquartile ranges of the error pooled over 9 participants and 15 trials across the five sessions. As in Experiment 1, performance improved fast in session 1 and reached a relatively steady level after session 2. This is highlighted by the exponential fit to the data. The changes in the median error decreased from 1.68 cm (session 1), 0.51 cm (session 2), 0.35 cm (session 3), 0.07 cm (session 4), to 0.14 cm (session 5). The following analyses pooled the data of sessions 2 to 5 where performance had converged towards what subjects regarded as their best solutions.

#### Pooled distributions

Returning to Figure 3B the histogram plotted in execution space pools all trials of all participants in sessions 2 to 5 onto a grid of 36x36 defined over the entire execution space. As can be seen, the data were distributed across a large range of velocities between 140 and 880 deg/s with the mode of the data distribution at 544 deg/s. This mode is between the maxima predicted by the tolerance hypothesis and the velocity hypothesis. To scrutinize whether individual subjects favored either one or the other strategy, we examined the individual distributions.

#### Individual distributions

Figure 6B shows the confidence ellipses for each participant calculated from the data of the four sessions (720 trials for each ellipse). The individuals' means were distributed across different velocities ranging between 240 to 775 deg/s with overlapping distributions. Only one participant's confidence ellipse came close to the peak of *E(R)* derived from Hypothesis 1. Similarly, only one participant's confidence ellipse enclosed the peak predicted by Hypothesis 2. However, it needs to be kept in mind that the gradient of *E(R)* across velocities was very small.

#### Hypothesis testing

**Figure 7:**
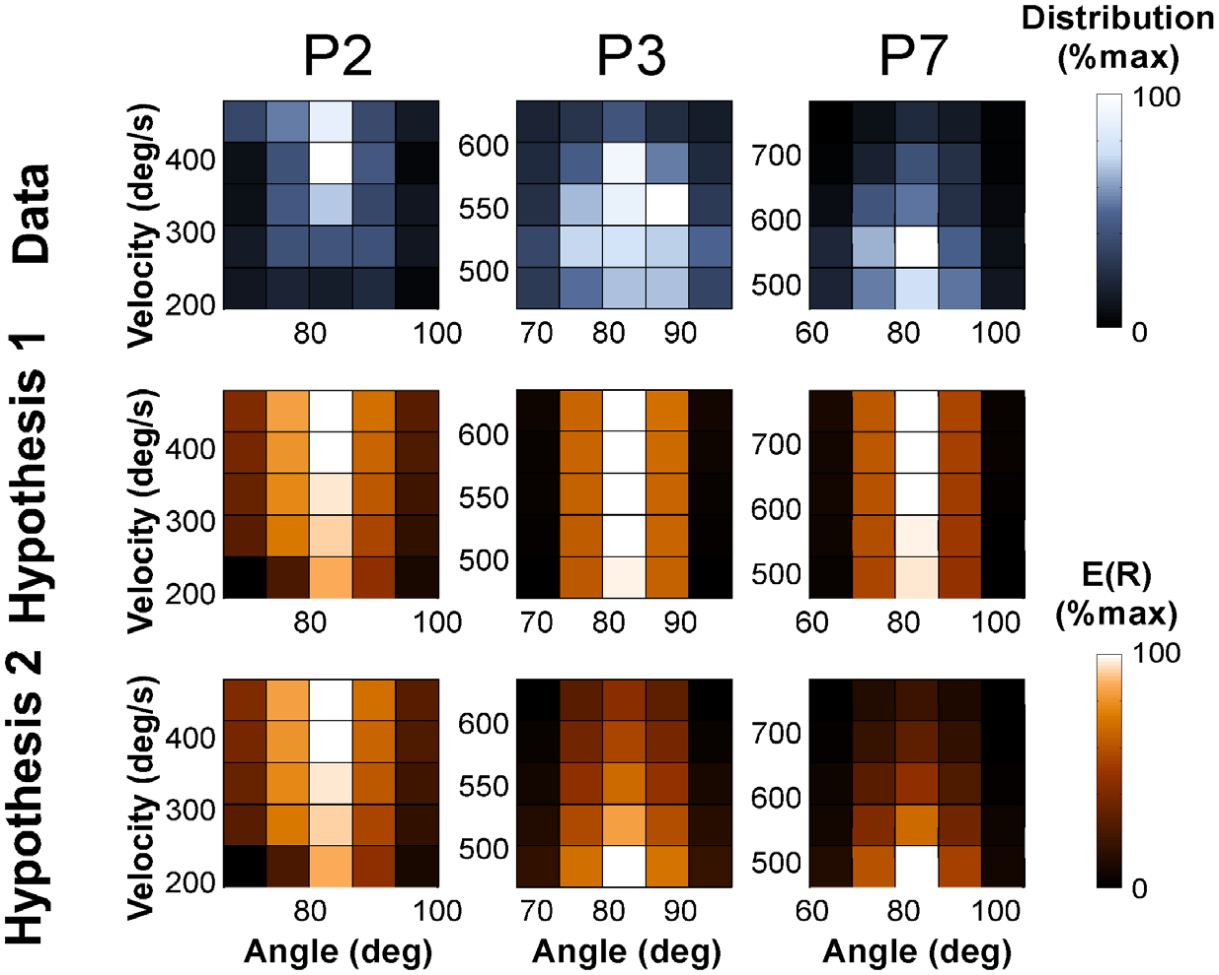
Two-dimensional histograms of two representative individuals' data and the corresponding hypothesized distributions for Experiment 2. The left panel shows the trial frequency, the right panel shows the expected result *E(R)* of Hypothesis 1, the right panel shows the predicted distribution of Hypothesis 2. As the units of the three distributions are different they were all normalized to the range between 0 and 1. Note that the black color codes the lowest value and should not be mistaken for the high-penalty regions in Figure 3.

We proceeded with finer-grained analyses that examined the distributions of each subject with respect to the two hypothesized distributions of *E(R)*. As for Experiment 1 the two hypotheses were recalculated based on each individual's distributions and all statistical tests were made locally depending on the individuals' chosen locations. Figure 7 shows three exemplary participants' histograms discretized into a 5x5 matrix in the execution space with the corresponding expected results *E(R)* for the two hypotheses. Participant 2 (with the second lowest mean velocity of 400 deg/s) shows a vertical data distribution biased to higher velocities consistent with Hypothesis 1 (participants were numbered in sequence of their mean velocity facilitating comparison with the individual data ellipses in Figure 6B). Participants 3 and 7 had significantly higher mean velocities and their distributions showed a tendency towards lower velocities, consistent with Hypothesis 2, yet not as pronounced as predicted.

Table 1 lists the results of these statistical comparisons. All 9 participants exhibited better fits for Hypotheses 2. However, the AIC was higher for every participant, giving support that the additional improvements of the log likelihood fit were insufficient to give significance. Hence, the results of all participants rejected Hypothesis 2.

#### Velocity-dependent variability

**Figure 8:**
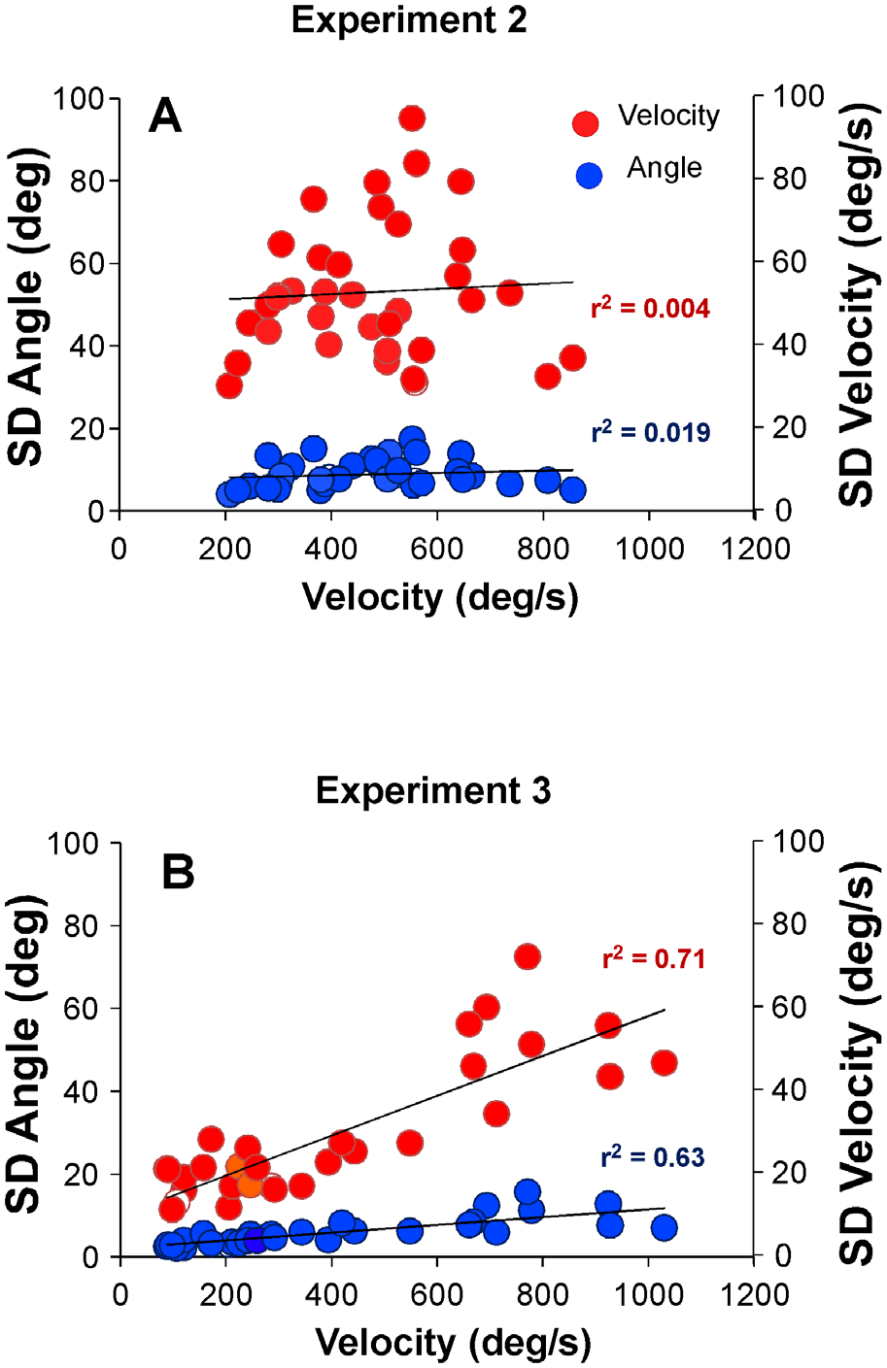
Standard deviations of velocity and angle plotted against their respective mean velocity. **A:** Data of all subjects in Experiment 2 (9 participants in 4 sessions with 3 blocks each). The linear regressions did not show any dependency of variability on the velocity. **B:** Data of Experiment 3 where subjects performed the same throwing movement but without a target. While standard deviations did not scale with increasing mean velocity in Experiment 2, velocity-dependent variability or noise was observed in this Experiment.

In the absence of support for Hypothesis 2, we examined the data whether there was indeed a cost to performances with higher velocities, i.e. higher variability associated with higher velocities. To this end, we calculated the mean and standard deviations of all velocities and angles in each of the 3 blocks for all 4 sessions of all subjects. Plotting these standard deviations of angle and velocity against their respective mean velocities revealed that the data did not show a velocity-dependent variability (Figure 8A). Neither variability of velocity, nor variability of angle showed the expected increase with mean velocity, as evidenced by the small and non-significant r^2^-values of the linear regressions (SDAngle: *r^2^* = 0.019, *p* = 0.427, SDVelocity: *r^2^* = .004, *p* = 0.723).

Given this unexpected result, Experiment 3 was added as a control experiment. For each of the 5 blocks of 25 trials per participant that were performed under the instruction to keep the velocity similar, the mean of velocity and angle and its standard deviations was calculated. Figure 8B shows the results for all six participants plotting standard deviations of velocity and angle against the mean velocity. The linear regressions were significant with *r^2^*-values of .71 (*p*<.0001) and .63 (*p*<.0001), showing that variability increased significantly with increasing velocity.

## Discussion

Given the many spatial and temporal scales of the sensorimotor system, it is not surprising that at the level of observed actions there is always variability. Different sources for this variability have been identified: Recording in single neurons in the cortex Churchland and colleagues demonstrated that fluctuations in neuronal activity in M1 and dorsal premotor cortex during movement preparation accounts for half of the observed variability in the velocity profiles of reaching trajectories [1]. Muscle physiological studies demonstrated that the signal-dependent magnitude of noise in isometric force production was induced by twitch amplitude and the recruitment order of motor neurons [24]. Other physiological underpinnings of variability are reviewed in [5], [7]. A behavioral study by van Beers and colleagues attributed the observed variability to the actual execution of the reaching movement arguing that the variability was a mixture of signal-dependent and signal-independent noise [3]. Taken together, the complex processes in the underlying substrate give rise to fluctuations in observed behavior that are never completely suppressed and, as it may be speculated, should not be suppressed.

### Variability Due to Task Redundancy

These reviewed studies on sources of variability discussed the presence or absence of variability in terms of its amplitude and generally implied a random structure. While it is beneficial if this noise amplitude is reduced, the nervous system has also found other ways to reduce undesired variability in the behavioral outcome. If a given task is redundant, one such way is to channel variability into directions that have little effect on the end result. For example, the linkage of joints in the arm may covary without necessarily affecting the outcome, as shown for example in pistol shooting [25], [26], [27], [28], dart throwing, Boule throwing [29] and basketball throwing [30]. Much experimental evidence has been accumulated for this phenomenon and covariance has been generalized as a signature of synergies [31]. Channeling of variability into “do-not-care” directions is also an important consequence of stochastic optimal feedback control as applied to motor tasks by Todorov and colleagues [32], [33].

Our previous studies have similarly shown covariation in the structure of variability, although our three-pronged approach differentiated between magnitude and anisotropy of the data distribution (*Noise* and *Covariation*). It also separated off *Tolerance*, the aspect that figured centrally in the present study [20]. Core to our task-based analysis is the distinction between execution and result space: by mapping executions into results, the layout and geometry of the results can be obtained. This not only offers a quantitative understanding of the zero-error solutions but also of their neighborhood and the curvature, i.e. their sensitivity to error. In previous work we introduced *Tolerance*, a concept that allows quantification of what is the optimal strategy for a given distribution or variability [19], [22]. The present study extended this work by developing *a priori* predictions where and how variability should be distributed if the nervous system chose error-tolerant solution strategies.

### Hypothesis 1: Subjects Maximize Error Tolerance

The first hypothesis was that in skilled performance actors are aware of the limited resolution in their control and take their variability into account when planning and executing a movement. This hypothesis was tested by calculating the expected result in a neighborhood around each solution, i.e. by quantifying the degree of tolerance of a given movement strategy. This approach differs from standard sensitivity analyses in linearized systems that assess the effects of small deviations from a single solution. Specifically, local linear stability analysis evaluates how (infinitesimally) small perturbations destabilize a solution; relaxation time provides a quantitative measure for how fast a system returns to the stable solution. However, such an approach is ignorant to the effects of slightly larger errors. Knowledge of an extended neighborhood, however, is important when the system is nonlinear and has discontinuities like the result space in the skittles task. Considering that in human performance perturbations or errors have a sizable variance and the result space is nonlinear as in our skittles task, it is appropriate to assess error sensitivity not only at a point, but in a neighborhood around a chosen solution (for discussion of such analyses in nonlinear systems see [34]). The present study presented an analysis that quantifies error-tolerant strategies by assessing an “area” of solutions determined from the actual variability of subjects and evaluated the expected performance for such variability.

Results of two different task variations supported that subjects seek error-tolerant strategies. In Experiment 1 the data distributions of all nine participants were best fitted by the predictions of Hypothesis 1. However, the results did not rule out that subjects also minimized velocity as the solutions with the highest Tolerance were close to solutions at relatively low velocities. Interestingly, some individuals' strategies were also close to solutions with high penalty for hitting the center post. These inter-individual strategies may reflect the individual's attitude to risk, a topic that has been investigated by [35].

The rationale and the results of our study are in overall accordance with a series of experiments by Trommershäuser, Maloney and colleagues [9], [36], [37]. Using a speed-accuracy pointing task where the target area was bounded by a penalty area (at different distances and with different penalties), the distribution of hits was examined with respect to the expected gain. Formalized in a decision theoretical framework where a gain function is optimized based on the weighted sum of the gain and the subject's inherent variability, the results showed systematic effects of the penalty on the distributions. The results therefore supported the conclusion that selection of a movement strategy is largely determined by the subject's inherent variability. In contrast to the present study, hitting success was binary (positive for the target area and negative for the penalty area) while our focus was on the continuous distance to the target, which was prerequisite to the sensitivity analysis central to our study. Importantly, in these experiments the reward or penalty was endpoint accuracy that was directly visible to the subject on a monitor. In the skittles task the variables at release were not visible and important variables needed to be learnt via proprioceptive information across repeated trials, not itself visible to the actor. Further, the solution manifold and the sensitivity of its neighborhood are highly nonlinear and it is unlikely that performers have a priori an internal model of the result space.

### Hypothesis 2: Subjects Prefer Solutions with Minimum Velocity

Hypothesis 2 was formulated based on the widely accepted findings that performance variability scales with movement speed such that performance at higher velocities is more variable [38], [39], [40]. Assuming movement velocity reflects the amplitude of the motor control signal, this observation can be generalized that variability increases with signal strength and velocity. Physiologically, this behavioral observation has been related to the organizational properties of the motor unit pool such as recruitment order and twitch amplitudes [24]. In addition, it has also been commonly argued that subjects aim to minimize energy, either mechanical or metabolic. In the case of skittles, it may be hypothesized that subjects seek throws with minimum momentum of the arm movement. Taking these arguments together the hypothesis can be formulated that subjects should seek solutions with the lowest possible velocities [6], [41].

This alternative hypothesis was tested in Experiment 2 that was designed to explicitly dissociate between the two hypotheses. The target configuration was modified to create an execution space that permitted a large range of velocities to achieve successful hits where Hypothesis 2 clearly predicted the lowest possible velocity as the preferred strategy. In contrast, error tolerance showed a maximum at the highest velocities, although the gradient of *E(R)* across the higher velocities was relatively small. The individual subjects' distributions did not provide support for Hypothesis 2 and the subject averages and confidence ellipses extended over a large range of velocities. Consistent with this finding, analysis of velocity-dependent variability revealed that across subjects the variability did not increase with mean velocity.

### Velocity-Dependent Variability and Redundancy

To further scrutinize the apparent absence of velocity-dependent scaling of variability, an additional experiment was conducted to test whether this finding was due to the goal-oriented nature or the redundancy of the task. We speculated that if motor solutions cluster along the solution manifold this may obscure the otherwise reported increase in variability with movement velocity. In Experiment 3 subjects executed the same movement but did not aim for a target skittle. Hence, there was no solution manifold constraining the actions. This result highlighted that task redundancy introduces a solution manifold that presents a constraint that may suppress the velocity-dependent variability. This result is important as it qualifies the frequently adopted general assumption that variability and noise increases with signal amplitude.

As a final comment, it should be pointed out that our approach is completely confined to the kinematic level of task performance. Limb dynamics or other biomechanical considerations are not taken into explicit consideration. This is justified on two counts: First, the skittles task is performed by only a single-joint rotation in the horizontal plane where the rotating joint is fixed to the axle of the lever arm. Hence, neither intersegmental torques in the executing arm nor gravitational influences are of immediate concern. Second, much research on upper limb movements has provided evidence that endpoint trajectories may be planned in kinematic extrinsic coordinates [42], [43]. The analysis uses angular rotations of the manipulandum as defined in extrinsic coordinates with respect to the screen. That said, we did not address potential biomechanical considerations that arise from the positioning of the body with respect to the manipulandum or with what joint angles the angular rotations were executed. Subjects were told to position themselves in the most comfortable position, both with respect to any biomechanical concerns and with respect to optimal vision of the screen. At present, we refrained from including such additional considerations as these would have required additional motion capture.

In summary, two experiments examined a virtual throwing task and presented an analysis that provided an a priori hypothesis about which strategies actors should employ if they optimized error-tolerance. Analysis of the relation between the variability in execution to the result of the task performance revealed that actors not only decreased their motor variability in execution variables that mattered for the success of the task. The findings also gave strong support that subjects were sensitive to their motor variability and preferred strategies that optimized error tolerance.

## Supporting Information

Text S1 Calculation of Expected Results for Hypotheses 1 and 2.
(DOCX)

## References

[1] Churchland MM, Afshar A, Shenoy KV (2006). A central source of movement variability.. Neuron.

[2] van Beers RJ (2009). Motor learning is optimally tuned to the properties of noise.. Neuron.

[3] van Beers RJ, Haggard P, Wolpert DM (2004). The role of execution noise in movement variability.. J Neurophysiol.

[4] Osborne LC, Lisberger SG, Bialek W (2005). A sensory source for motor variation.. Nature.

[5] Faisal AA, Selen LP, Wolpert DM (2008). Noise in the nervous system.. Nature Rev Neuroscience.

[6] Harris CM, Wolpert DM (1998). Signal-dependent noise determines motor planning.. Nature.

[7] Bays PM, Wolpert DM (2007). Computational principles of sensorimotor control that minimize uncertainty and variability.. J Neurophysiol.

[8] Trommershäuser J (2009). Biases and optimality of sensory-motor and cognitive decisions.. Prog Brain Res.

[9] Trommershäuser J, Maloney LT, Landy MS (2008). Decision making, movement planning and statistical decision theory.. Trends Cogn Sci.

[10] van Beers RJ, Baraduc P, Wolpert DM (2002). Role of uncertainty in sensorimotor control.. Philos Trans R Soc Lond A.

[11] Valero-Cuevas F, Venkadesan M, Todorov E (2009). Structured variabilty of muscle activations supports the minimla intervention principle of motor control.. J Neurophysiol.

[12] Liu D, Todorov E (2007). Evidence for the flexible sensorimotor strategies predicted by optimal feedback control.. J Neurosci.

[13] Todorov E (2004). Optimality principles in sensorimotor control.. Nat Neurosci.

[14] Ronsse R, Wei K, Sternad D (2010). Optimal control of cyclical movements: the bouncing ball revisited.. J Neurophysiol.

[15] Nagengast AJ, Braun DA, Wolpert DM (2010). Risk-sensitive optimal feedback control accounts for sensorimotor behavior under uncertainty.. PLoS Comput Biol.

[16] Latash ML, Scholz JP, Schöner G (2002). Motor control strategies revealed in the structure of motor variability.. Exer Sport Sci Rev.

[17] Scholz JP, Schöner G (1999). The uncontrolled manifold concept: identifying control variables for a functional task.. Exp Brain Res.

[18] Sternad D, Park S, Müller H, Hogan N (2010). Coordinate dependency of variability analysis.. PLoS Comput Biol.

[19] Müller H, Sternad D, Sternad D (2009). Motor learning: Changes in the structure of variability in a redundant task.. Progress in Motor Control - A Multidisciplinary Perspective.

[20] Cohen RG, Sternad D (2009). Variability in motor learning: relocating, channeling and reducing noise.. Exp Brain Res.

[21] Müller H, Sternad D (2003). A randomization method for the calculation of covariation in multiple nonlinear relations: Illustrated at the example of goal-directed movements.. Biol Cybern.

[22] Müller H, Sternad D (2004). Decomposition of variability in the execution of goal-oriented tasks – Three components of skill improvement.. J Exp Psychol Hum Percept Perform.

[23] Sutton RS, Barto AG (1998). Reinforcement learning: An introduction..

[24] Jones KE, Hamilton AF, Wolpert DM (2001). Sources of signal-dependent noise during isometric force production.. J Neurophysiol.

[25] Arutyunyan GH, Gurfinkel VS, Mirskii ML (1968). Investigation of aiming at a target.. Biophysics.

[26] Cusumano JP, Cesari P (2006). Body-goal variability mapping in an aiming task.. Biol Cybernetics.

[27] Scholz JP, Schöner G, Latash ML (2000). Identifying the control structure of multijoint coordination during pistol shooting.. Exp Brain Res.

[28] Müller H, Loosch E (1999). Functional variability and an equifinal path of movement during targeted throwing.. J of Hum Mov Studies.

[29] Dupuy MA, Mottet D, Ripoll H (2000). The regulation of release parameters in underarm precision throwing.. J Sports Sci.

[30] Kudo K, Tsutsui S, Ishikura T, Ito T, Yamamoto Y (2000). Compensatory correlation of release parameters in a throwing task.. J Mot Behav.

[31] Latash ML (2008). Synergy..

[32] Todorov E, Jordan MI (2002). Optimal feedback control as a theory of motor coordination.. Nat Neurosci.

[33] Todorov E (2005). Stochastic optimal control and estimation methods adapted to the noise characteristics of the sensorimotor system.. Neural Comput.

[34] Guckenheimer J, Holmes P (1983). Nonlinear oscillations, dynamical systems, and bifurcations of vector fields..

[35] Seydell A, McCann BC, Trommershäuser J, Knill DC (2008). Learning stochastic reward distributions in a speeded pointing task.. J Neurosci.

[36] Trommershäuser J, Gepshtein S, Maloney LT, Landy MS, Banks MS (2005). Optimal compensation for changes in task-relevant movement variability.. J Neurosci.

[37] Trommershäuser J, Maloney LT, Landy MS (2003). Statistical decision theory and trade-offs in the control of motor responses.. Spat Vis.

[38] Worringham CJ (1991). Variability effects on the internal structure of rapid aiming movements.. J Mot Behav.

[39] Worringham CJ, Newell KM, Corcos DM (1993). Predicting motor performance from variability measures.. Variability and motor control.

[40] Schmidt RA, Zelaznik H, Hawkins B, Frank JS, Quinn JT (1979). Motor-output variability: a theory for the accuracy of rapid notor acts.. Psych Review.

[41] Hamilton AF, Wolpert DM (2002). Controlling the statistics of action.. J Neurophysiol.

[42] Flanagan JR, Rao AK (1995). Trajectory adaptation to a nonlinear visuomotor transformation: evidence of motion planning in visually perceived space.. J Neurophysiol.

[43] Gordon J, Ghilardi MF, Ghez C (1994). Accuracy of planar reaching movements: I. Independence of direction and extent.. Exp Brain Res.

