# Supplementary material for: Neuromotor Noise, Error Tolerance and Velocity-Dependent Costs in Skilled Performance
Source: PLoS Comput Biol. 2011 Sep 22;7(9):e1002159. doi: 10.1371/journal.pcbi.1002159 (PMC3178634; doi:10.1371/journal.pcbi.1002159)
Supplement: Text S1 — Calculation of Expected Results for Hypotheses 1 and 2. (DOCX) [file pcbi.1002159.s001.docx]

**S1: Supporting Information**

**Calculation of Expected Result for Hypotheses 1 and 2**

For the simulation of the predictions for the two hypotheses, the execution space was divided into 360*360 bins over all possible angles from –180 to +180 deg and velocities between 0 and 1000 deg/s. The upper boundary for velocity was experimentally determined as the upper limit that subjects could reach. The first step was to evaluate the error tolerance *T(R)* for each bin
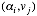
, with *i* = 1, 2… 360, *j* = 1, 2 ... 360. The second step was to transform these predictions into expected results *E(R)* or the predicted probability with which participants would chose this solution.

Before calculating *T(R)* the results or errors
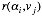
 were transformed to convert the penalty function into a reward function with post hits defined as zero
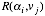
:


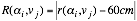


where
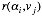
 was the error between the target and the ball trajectory for each throw (*a_i_* ,*v_j_*). (The initially assigned error for the posthit was 60 cm.)

For each
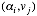
 a matrix was defined of a size that ranged over plus/minus one standard deviations of *α* and *v*. For the subjects’ variability observed in Experiment 1, this translated into a 12x15 matrix, for Experiment 2, this was equivalent to a 9x25 matrix. The probability distribution
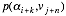
 was defined as:

where *pdf* is the probability density function of the bivariate normal distribution with mean
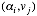
 and standard deviation (*SDα, SDv*). The indices *k* and *n* for Experiment 1 were: *k* = -7 to 7, *n* = -11 to 11; for Experiment 2: *k* = -4 to 4, *n* = -12 to 12.

The error tolerance at each
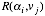
 was defined as


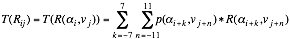


These calculations for *T(R_ij_)* were performed for all 360x360 grid points. When the weight matrix transgressed the boundary of the execution space, the outside cells of the weight matrix were eliminated and only the remaining cells were included in the computation. These calculations for *T(R_ij_)* were equivalent to the algorithm for expected utility in decision theory, with
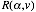
 representing the utility or cost function [1,2].

In a second step *T(R_ij_)* was submitted to the softmax function to obtain an estimate of the probability that participants would chose this value. This expected result *E(R_ij_)* was calculated for each cell as


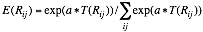


where *a* was chosen from the best likelihood fit of *E(R)* with the data pooled over all participants.

In Experiment 2 the softmax transformation was extended to include a term that accounted for the velocity, weighted by *b*:


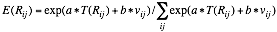


As in Experiment 1, the two constants *a* and *b* were obtained from optimizing the likelihood fit to the pooled data from all participants.

1. Berger JO (1985) Statistical decision theory and Bayesian analysis. New York: Springer.

2. Trommershäuser J, Maloney LT, Landy MS (2003) Statistical decision theory and trade-offs in the control of motor responses. Spatial Vision 16: 255-275.
